# Gynecologic cancer clinical practice guidelines in Korea and current issues

**DOI:** 10.4069/kjwhn.2022.05.10

**Published:** 2022-06-09

**Authors:** Jeeyeon Kim, Joo-Hyuk Son, Tae-Wook Kong, Suk-Joon Chang

**Affiliations:** Division of Gynecologic Oncology, Department of Obstetrics and Gynecology, Ajou University School of Medicine, Suwon, Korea

## Introduction

The most common gynecologic cancers in the world are ovarian cancer, endometrial cancer, and cervical cancer; these are also the most prevalent gynecologic cancers in Korea. According to the Korean Central Cancer Registry, these three types of cancer accounted for 6.7% of Korea’s cancer mortality in 2017, of which cervical cancer and ovarian cancer were the eighth and ninth most common causes of death, respectively [1]. From 1999 to 2017, the cumulative total number of patients with these three types of cancer was 134,863. The incidence of ovarian cancer and endometrial cancer is increasing annually, whereas that of cervical cancer is decreasing because of the National Cervical Cancer screening program and prophylactic human papillomavirus (HPV) vaccination [2]. Clinical guidelines for gynecologic cancers have been developed and published by all national groups and regularly updated based on recent evidence. In Korea, the Korean Society of Gynecologic Oncology practice guidelines are published periodically; here, we would like to introduce the latest version of these gynecologic cancer treatment guidelines briefly, along with recent findings.

## Ovarian cancer

Although the number of ovarian cancer patients is increasing globally, the incidence rate in Korea has remained stable. Most ovarian cancer patients are diagnosed at advanced stages and, despite proper treatment, ovarian cancer has a high mortality rate. Therefore, early diagnosis and treatment are very important. Although the incidence is relatively stable, there has been no significant improvement in survival outcomes for ovarian cancer patients.

It is known that nulliparity and first delivery after 35 years are risk factors for ovarian cancer. Moreover, *BRCA1* or *BRCA2* mutations and a familial history of conditions such as hereditary nonpolyposis colorectal cancer also increase the risk of ovarian cancer; therefore, *BRCA* genetic testing is recommended for all ovarian cancer patients [3]. Many studies have shown that in high-risk patients, risk-reducing salpingo-oophorectomy can reduce the risk of ovarian cancer, though it does not decrease the risk of peritoneal cancer [4].

The standard treatment for ovarian cancer is taxane/platinum chemotherapy after optimal cytoreductive surgery. The complete staging operation includes hysterectomy, bilateral salpingo-oophorectomy, omentectomy, bilateral pelvic and paraaortic lymph node dissection, multiple peritoneal biopsies (bilateral pelvic, paracolic gutter, and diaphragm biopsies), and peritoneal washing cytology [3]. Pelvic and paraaortic lymph node dissection can be performed based on the physician’s decision because it did not show clear evidence of a survival benefit [5]. For patients who want to preserve fertility, unilateral salpingo-oophorectomy may be considered for unilateral early-stage ovarian cancer. For advanced-stage ovarian cancer with extensive disease, neoadjuvant chemotherapy followed by interval debulking surgery can be done to reduce the tumor burden [6]. According to a recent randomized trial, ovarian cancer patients who received hyperthermic intraperitoneal chemotherapy after interval cytoreductive surgery showed more favorable outcomes in terms of progression-free and overall survival than ovarian cancer patients who received only surgery [7].

Only stage IA and IB patients do not need adjuvant treatment such as chemotherapy; in these patients, only follow-up is required. Other than these patients, all ovarian cancer patients must receive taxane/platinum chemotherapy. Bevacizumab can be used as the initial taxane/platinum regimen, as well as for subsequent maintenance therapy; and it has been proven to be beneficial by improving survival outcomes. Moreover, in therapy for recurrent cases, chemotherapy including bevacizumab can be used for platinum-sensitive cases [3]. Because of its proven therapeutic effect, bevacizumab has been approved for ovarian cancer treatment in Korea since 2015.

Interest has recently emerged in using poly (ADP ribose) polymerase (PARP) inhibitors for maintenance therapy. This option is considered in cases of *BRCA*-related ovarian cancer, especially in platinum-sensitive recurrent ovarian cancer with a *BRCA* mutation. Multiple studies have proven its efficacy, and PARP inhibitors have shown prolonged progression-free survival and overall survival, providing a median overall survival benefit of 12.9 months in patients with platinum-sensitive, relapsed ovarian cancer and a *BRCA1/2* mutation [8,9]. In Korea, while olaparib (Lynparza, AstraZeneca, Cambridge, UK) has been approved as maintenance for primary and secondary ovarian cancer starting from October 2021, niraparib (Zejula, GlaxoSmithKline company, London, UK) is still restricted to only patients with *BRCA* mutations [10].

## Endometrial cancer

Compared with western countries, the incidence of endometrial cancer in Korea is quite low. However, according to the Korea Central Cancer Registry, the prevalence of endometrial cancer has increased more rapidly in recent years, owing to an increase in the obesity rate caused by following a westernized lifestyle [2]. Moreover, the low birth rate in Korea also influences the increasing incidence of endometrial cancer because parity may reduce the risk of endometrial cancer [2]. Even though the prevalence of endometrial cancer is increasing, most patients are diagnosed at an early stage; therefore, endometrial cancer has a more favorable prognosis than other gynecologic cancers [11].

Most endometrial cancer patients visit the hospital because of abnormal vaginal bleeding. Once endometrial cancer is suspected, the diagnosis is confirmed by checking for risk factors (such as diabetes, obesity, tamoxifen use, estrogen use, and genetic factors) and conducting an endometrial biopsy if suspicious endometrial abnormalities are found on ultrasonography. Other imaging modalities including computed tomography (CT), magnetic resonance imaging (MRI), and positron emission tomography (PET)-CT scans can be used for preoperative staging and treatment plans.

The standard treatment for early-stage (stages I and II) endometrial cancer is total abdominal hysterectomy and bilateral salpingo-oophorectomy with or without lymphadenectomy, which can be done laparoscopically. Lymphadenectomy in patients with early endometrial cancer is still under discussion, as whether it improves survival rates has been a matter of debate. Recent studies have shown that lymphadenectomy in early-stage endometrial cancer itself has no survival benefit over procedures without lymphadenectomy. Therefore, lymphadenectomy can be omitted in patients with very low-risk endometrial cancer [11]. Oral progestin therapy can be considered in grade 1 endometrioid adenocarcinoma patients who strongly want to maintain fertility. Additionally, adjuvant therapy is needed in some endometrial cancer patients. Except for stage IA grade 1 patients, other early endometrial cancer patients also need additional treatment such as pelvic radiation therapy or brachytherapy; this decision is made based on patients’ risk factor and grade.

For advanced-stage endometrial cancer patients, a staging operation with adjuvant therapy is advised. After surgery, concurrent or sequential combined chemotherapy and radiotherapy are recommended [11].

## Cervical cancer

Globally, cervical cancer is the fourth most common cancer in women in terms of its prevalence and mortality rate. However, in most developed countries, both the prevalence and mortality rate are decreasing owing to screening tests and prophylactic HPV vaccination [12]. On the contrary, in developing countries, the prevalence and mortality rate remain high, contributing to more than 85% of the global prevalence [12]. The incidence of cervical cancer in Korea is decreasing, whereas the incidence of carcinoma in situ is increasing in all ages owing to early diagnosis and treatment [2]. HPV infection is known to be the most important risk factor for cervical cancer.

Although there are some differences between papers, the HPV infection rate in Korea is reported to be about 10% to 15% [2]. In cases of early cervical cancer (stage I or stage II), the cure rate is 80% to 90% because of effective treatment such as surgery or concurrent chemoradiation therapy. The cure rate in cases of stage III and recurrence is 60%, even with proper treatment, and the prognosis is known to be poor [2]. Like other gynecologic cancers, cervical cancer usually has no symptoms in the early stage, but in later stages, it is accompanied by symptoms such as increased vaginal discharge and vaginal bleeding. Cytology and HPV tests are effective in screening for cervical cancer, along with colposcopy-guided biopsies to improve the accuracy of the diagnosis. After the biopsy confirmation, imaging tests such as MRI and PET-CT are also performed; and cystoscopy and colonoscopy can be performed, especially when bladder and colon invasion is suspected. With all the above information, each patient’s International Federation of Gynecology and Obstetrics stage can be diagnosed, and proper treatment can be performed according to each stage. The squamous cell carcinoma (SCC) antigen is a useful serologic tumor marker, and the SCC antigen level is related to the disease burden. Therefore, this marker should be measured before treatment and during follow-up to predict treatment response and recurrence.

In 2018, a prospective phase III randomized controlled trial using the laparoscopic approach to carcinoma of the cervix reported that minimally invasive surgery was associated with statistically significant poorer overall survival and disease-free survival rates compared with open surgery in patients with early-stage cervical cancer [13].

Furthermore, the addition of bevacizumab to combination chemotherapy was associated with an improvement of 3.7 months in median overall survival and a higher response rate [14]. Therefore, bevacizumab can now be used for recurrent or persistent cervical cancer to improve survival outcomes [14], especially since it was covered by insurance in Korea in 2015.

## Implications

In Korea, the incidence rate of gynecologic cancers increased annually by 2.76% until 2017 [2]. HPV vaccination and national screening testing for cervical cancer are important parts of cervical cancer prevention. Hence, all medical teams should strive to inform the public about the importance of cancer prevention and routine screening tests. For endometrial cancer, patients should not only be informed about the treatment of the disease, but also about lifestyle modification and obesity. Lastly, ovarian cancer screening testing and early detection have not been effective to date. Therefore, more research should be done on prevention, early detection, and the possibility of a familial history. We should educate patients about genetic testing, which can provide more treatment options for ovarian cancer, and provide information about risk-reducing surgery and regular surveillance testing for *BRCA*-positive ovarian cancer patients.

Nurses’ and the healthcare team’s level of understanding of appropriate clinical guidelines and continued interest in the latest gynecologic cancer issues can have a positive impact on the development of gynecologic cancer prevention and treatment protocols. More clinical research is needed to reduce the incidence of gynecologic cancer and to increase the survival rate through early diagnosis and proper treatment.
